# Exposure of bovine oocytes and embryos to elevated non-esterified fatty acid concentrations: integration of epigenetic and transcriptomic signatures in resultant blastocysts

**DOI:** 10.1186/s12864-016-3366-y

**Published:** 2016-12-08

**Authors:** K. L. J Desmet, V. Van Hoeck, D. Gagné, E. Fournier, A. Thakur, A. M. O’Doherty, C. P. Walsh, M. A. Sirard, P. E. J. Bols, J. L. M. R. Leroy

**Affiliations:** 1 Laboratory of Veterinary Physiology and Biochemistry, Department of Veterinary Sciences, Faculty of Pharmaceutical, Biomedical and Veterinary Sciences, University of Antwerp, Wilrijk, Belgium; 2 Centre de Recherche en Biologie de la Reproduction (CRBR), Département des Sciences Animales, Université Laval, Québec, Canada; 3 British Columbia Cancer Agency, University of British Columbia, Vancouver, Canada; 4 School of Agriculture and Food Science, University College Dublin, Dublin, Ireland; 5 Centre for Molecular Biosciences, School of Biomedical Sciences, University of Ulster, Coleraine, UK

**Keywords:** Oocyte, Embryo, Fertility, Free fatty acids, Maternal metabolism, DNA methylation, Epigenetics

## Abstract

**Background:**

Metabolic stress associated with negative energy balance in high producing dairy cattle and obesity in women is a risk factor for decreased fertility. Non-esterified fatty acids (NEFA) are involved in this pathogenesis as they jeopardize oocyte and embryo development. Growing evidence indicates that maternal metabolic disorders can disturb epigenetic programming, such as DNA methylation, in the offspring. Oocyte maturation and early embryo development coincide with methylation changes and both are sensitive to adverse environments. Therefore, we investigated whether elevated NEFA concentrations affect establishment and maintenance of DNA methylation in oocytes and embryos, subsequently altering transcriptomic profiles and developmental competence of resultant blastocysts.

**Results:**

Bovine oocytes and embryos were exposed to different NEFA concentrations in separate experiments. In the first experiment, oocytes were matured in vitro for 24 h in medium containing: 1) physiological (“BASAL”) concentrations of oleic (OA), palmitic (PA) and stearic (SA) acid or 2) pathophysiological (“HIGH COMBI”) concentrations of OA, PA and SA. In the second experiment, zygotes were cultivated in vitro for 6.5 days under BASAL or HIGH COMBI conditions. Developmental competence was evaluated by assessing cleavage and blastocyst rate. Overall gene expression and DNA methylation of resultant blastocysts were analyzed using microarray. DNA methylation data were re-evaluated by pyrosequencing. HIGH COMBI-exposed oocytes and embryos displayed a lower competence to develop into blastocysts compared to BASAL-exposed counterparts (19.3% compared to 23.2% and 18.2% compared to 25.3%, respectively) (*P* < 0.05). HIGH COMBI-exposed oocytes and embryos resulted in blastocysts with altered DNA methylation and transcriptomic fingerprints, compared to BASAL-exposed counterparts. Differences in gene expression and methylation were more pronounced after exposure during culture compared to maturation suggesting that zygotes are more susceptible to adverse environments. Main gene networks affected were related to lipid and carbohydrate metabolism, cell death, immune response and metabolic disorders.

**Conclusions:**

Overall, high variation in methylation between blastocysts made it difficult to draw conclusions concerning methylation of individual genes, although a clear overview of affected pathways was obtained. This may offer clues regarding the high rate of embryonic loss and metabolic diseases during later life observed in offspring from mothers displaying lipolytic disorders.

## Background

Non-esterified fatty acid (NEFA) concentrations are a common feature of an impaired maternal metabolism, typically observed in cows suffering negative energy balance but also in obese and type-II diabetes patients. Earlier research has revealed that the fertility of females suffering from these metabolic disorders is compromised [1]. Awareness grows that NEFAs within the gamete’s and/or embryonic microenvironment may play a key role in causing this compromised fertility outcome [2]. Using a bovine model, we and others have previously shown that elevated NEFA concentrations during oocyte maturation and embryo culture are detrimental for embryonic development [1, 3]. Additionally, elevated NEFA levels in the oocyte’s microenvironment can affect gene expression, DNA methylation at imprinted genes and alter the phenotype of resultant blastocysts [4–6]. Lower cell numbers and increased apoptosis were observed in blastocysts after exposure of oocytes to elevated NEFA concentrations. Moreover, these blastocysts displayed reduced pyruvate, glucose and oxygen consumption, increased lactate consumption and altered amino acid metabolism compared to control blastocysts [4]. Notably, oocyte maturation under high NEFA concentrations significantly altered the expression of genes involved in establishing methylation patterns in the matured cumulus oocyte complex, as well as in resultant day 7.5 blastocysts [5, 7]. Cumulus cells from HIGH COMBI-exposed oocytes exhibited down-regulated expression of *DNMT3A* [7], which is involved in *de novo* methylation of cytosine residues at CpG sites in oocytes and early preimplantation embryos until embryonic genome activation [8, 9]. This finding also raises the possibility of altered methylation status due to exposure to adverse maternal metabolic conditions.

Genes regulating DNA methylation, e.g. *DNMTs*, are crucial for driving appropriate growth and differentiation in the developing embryo, especially during oocyte maturation and early embryo development [9–12]. In the oocyte, an increase in DNA methylation occurs during its growth with the highest level reached at the germinal vesicle stage [13, 14]. Following fertilization, DNA demethylation takes place in the zygote and continues in the subsequent cleavage stages [15]. Re-establishment of general DNA methylation patterns succeeds embryonic genome activation [16]. At the time of blastocyst formation, DNA remethylation is still an ongoing process that depends on blastocyst’s stage, gender and ratio of inner cell mass (ICM) versus trophectoderm (TE) [17]. During post-blastocyst development, cell differentiation occurs with the establishment of cell lineage-specific DNA methylation patterns [17, 18]. Taken together, it is now generally accepted that oocyte maturation and preimplantation embryo development are sensitive windows for epigenetic reprogramming [19].

Biochemical changes in the oocytes microenvironment, associated with maternal metabolic disturbances [20–22], may result in alterations to the epigenome that, in turn, can compromise fertility or even result in persistent changes during fetal development or become visible after birth [23]. This concept is known as ‘Developmental Origins of adult Health and Disease’ or DOHAD, first described by Barker et al. [24]. In this context, Ge et al. [25] reported that maternal diabetes mellitus can affect DNA methylation imprinting in murine oocytes. Jungheim et al. [26] showed that mouse embryos, exposed to elevated palmitic acid concentrations, presented an altered embryonic metabolism and development, with lasting adverse effects on growth patterns in offspring suggested to be associated with aberrant epigenetic programming. Assisted reproduction techniques have also been shown to influence epigenetic mechanisms during oocyte maturation and preimplantation embryo culture, leading to, for example, large offspring syndrome in ruminants [27] or a higher risk for metabolic syndrome in children born after in vitro fertilisation [28, 29].

We hypothesize that elevated NEFA concentrations during oocyte maturation or embryo culture, the most sensitive windows during which DNA methylation reprogramming occurs, jeopardize embryo developmental competence by impacting the blastocysts transcriptome and DNA methylation signatures. Considering the dynamic properties of DNA methylation at blastocyst stage [17], we propose to focus first on overall affected pathways rather than on gene-specific changes. Therefore, the aim of the present study was to examine the effect of elevated NEFA concentrations during 24 h of oocyte maturation or during 6.5 days of embryo culture on:

1. transcriptome signatures of resultant day 7.5 blastocysts and, in particular, the expression of genes involved in epigenetic pathways using the bovine EmbryoGENE microarray technique,
2. whole-genome DNA methylation signatures of resultant day 7.5 blastocysts using the EmbryoGENE DNA Methylation Array (EDMA, http://emb-bioinfo.fsaa.ulaval.ca/).

## Methods

### Experimental design

The types and concentrations of free fatty acids used in this study are based on bovine in vivo studies in serum and follicular fluid during a period of negative energy balance [30] and have been reported for in vitro use before by Van Hoeck et al. [4]. Valckx et al. [22] also observed that palmitic (PA, C16:0), stearic (SA, C18:0) and oleic acid (OA, C18:1) are the most abundant NEFAs in human follicular fluid of obese patients.

Two experiments were set up to characterize the developmental capacity and the transcriptomic and epigenomic profile of the resultant day 7.5 blastocysts.

In the first (in vitro maturation or IVM) experiment, the effect of elevated NEFA exposure during oocyte maturation (24 h) on embryo development, gene expression and DNA methylation profiles was evaluated by exposing oocytes to the following conditions:

1. BASAL: physiological NEFA concentrations (72 μM total NEFA containing 28 μM SA, 23 μM PA, and 21 μM OA).
2. HIGH COMBI: a combination of elevated NEFA concentrations equivalent to those measured in the follicular fluid during severe lipolytic conditions (425 μM total NEFA, containing 75 μM SA, 150 μM PA, and 200 μM OA).

In the second (in vitro culture or IVC) experiment, the effect of elevated NEFA exposure during embryo culture (6.5 days) on embryo development, gene expression profile and DNA methylation was evaluated by exposing embryos to the following conditions:

1. BASAL: physiological NEFA concentrations (72 μM total NEFA containing 28 μM SA, 23 μM PA, and 21 μM OA).
2. HIGH COMBI: a combination of elevated NEFA concentrations equivalent to those measured in serum under high lipolytic conditions (720 μM total NEFA, containing 280 μM SA, 230 μM PA, and 210 μM OA).

In order to evaluate the effects of elevated NEFA exposure on embryo development, cleavage rate (48 h p.i.) and blastocyst yield (7.5 days p.i.) were recorded in both experiments. Evaluation of the gene expression profile of blastocysts originating from NEFA-exposed oocytes was previously performed by Van Hoeck et al. [5]. In the IVC experiment, 4 replicates were produced to investigate overall gene expression (using a total of 648 oocytes equally distributed between treatments). Resultant day 7.5 blastocysts were snap frozen for genome-wide analysis of transcription (80 blastocysts, equally collected between treatments in 4 replicates). Blastocysts from NEFA-exposed oocytes (IVM experiment) and embryos (IVC experiment) were produced for genome-wide analysis of DNA methylation profiles. A total of 1039 and 1412 oocytes were used respectively, equally distributed between treatments in 4 replicates. Resultant day 7.5 blastocysts were snap frozen for subsequent DNA methylation analysis (80 blastocysts in each experiments, equally allocated between treatments in 4 replicates). Results obtained from the EDMA analysis were assessed using pyrosequencing of 4 independent replicates of blastocysts in the IVM and IVC experiment (879 and 809 oocytes respectively, equally distributed between treatments). Pools of 10 blastocysts were snap frozen per treatment and per replicate in both experiments.

### Preparation of NEFA treatments

Media containing NEFAs were prepared as previously described by Van Hoeck et al. [4]. SA, PA and OA were dissolved in a stock solution of pure ethanol at different concentrations according to the experiment and treatment. In the IVM experiment, NEFA stocks were prepared at concentrations of 28, 23 and 21 mM for the BASAL treatment and 25, 150 and 200 mM for the HIGH COMBI treatment. In the IVC experiment, NEFA stocks were prepared at concentrations of 28, 23 and 21 mM for the BASAL treatment and 112, 230 and 210 mM for the HIGH COMBI treatment, respectively. The final medium was supplemented with fatty acid-free 0.75% bovine serum albumin (BSA) in order to improve NEFA solubility.

### In vitro embryo production

In vitro embryo production procedures were performed as previously described by Van Hoeck et al. [4], using immature oocytes retrieved from bovine ovaries collected from a local abattoir within 2 h of slaughter. Briefly, grade I cumulus oocyte complexes (COCs) were matured in groups of 50–60 in 500 μl serum-free maturation medium containing TCM199 supplemented with fatty acid-free 0.75% BSA, 0.4 mM glutamine, 0.2 mM sodium pyruvate, 0.1 mM cysteamine, 50 mg/ml gentamycin and murine epidermal growth factor (mEGF, 20 ng/ml) for 24 h in humidified air with 5% CO_2_ at 38.5 °C. In the IVM experiment, oocytes were randomly divided in equal groups between treatment-specific maturation media. In the IVC experiment, oocytes were matured in serum-free maturation medium as mentioned above. After IVM, COCs were co-incubated in groups of 100–120 with spermatozoa at a final concentration of 10^6^/ml for 22 h at 38.5 °C in 500 μl fertilization medium (containing 114 mM NaCl, 3.1 mM KCl, 0.3 mM Na_2_HPO_4_, 2.1 mM CaCl_2_-2H_2_O, 0.4 mM MgCl_2_-6H_2_O, 25 mM bicarbonate, 1 mM pyruvate, 36 mM lactate, 2 μL/ml phenol red, 6 mg/mL fatty acid-free BSA, 50 μg/mL gentamycin and 0.72U/mL heparin) in a humidified 5% CO_2_ incubator. Presumptive zygotes were cultured in groups of 25 ± 4. During the IVM experiment, the presumptive zygotes were cultured in 50 μl droplets of mSOF medium with a mineral oil overlay (modular incubator: 38.5 °C, 5% CO_2_, 5% O_2_ and 90% N_2_) until the day of analysis. During the IVC experiment, the presumptive zygotes were incubated in a reduced surface 96-well dish containing 75 μl medium without mineral oil overlay (modular incubator: 38.5 °C, 5% CO_2_, 5% O_2_ and 90% N_2_). The mSOF medium contained 108 mM NaCl, 7.2 mM KCl, 1.2 mM KH_2_PO_4_, 0.8 mM MgSO_4_.7H_2_O, 0.6 mM sodium lactate, 25 mM NaHCO_3_, 0.0266 mM phenol red, 0.73 mM sodium pyruvate, 1.78 mM CaCl_2_.2H_2_O, 0.34 mM trisodium citrate, 2.755 mM myoinositol, 3% *v/v* BME 50x, 1% *v/v* MEM 100x, 0.4 mM glutamine, 5% fetal bovine serum and 50 μg/mL gentamycin. For the IVC experiment, NEFAs were added to the mSOF medium at concentrations according to the treatment.

### Transcriptomic analysis

Gene expression analysis using the bovine EmbryoGENE microarray slides was performed as previously described by Cagnone et al. [31]. Total RNA from pools of 10 blastocysts (pools of normal and expanded blastocysts, equally distributed per treatment and per replicate) was extracted and purified using the PicoPure RNA Isolation Kit (ThermoFisher Scientific, Ottawa, Ontario). After DNase treatment (Qiagen, Toronto, Canada), quality and concentration of the extracted RNA were analysed using a bioanalyzer (Agilent, Diegem, Belgium). All extracted samples showed good quality with an RNA integrity number >7.5. In total, 42,242 total probes were covered including 21,139 known reference genes, 9,322 probes for novel transcribed regions, 3,677 alternatively spliced exons, 3,353 39-tiling probes, and 3,723 control probes.

### Quantification of DNA methylation patterns

DNA methylation analysis using the bovine EmbryoGENE DNA Methylation Array (EDMA) was performed as previously described by Shojaei et al. [32]. Genomic DNA and total RNA were extracted from pools of 10 blastocysts (pools of normal and expanded blastocysts, equally distributed per treatment and per replicate). The microarray covered a total of 414,566 probes surveying 20,355 genes and 34,379 CpG islands. Data handling was conducted using a built-in pipeline to perform pre-processing (data quality control and normalization) and analysis steps (statistical analysis and data sorting) [32] (http://emb-bioinfo.fsaa.ulaval.ca/).

### Targeted DNA methylation analysis using pyrosequencing

DNA was isolated from a pool of 10 blastocysts (pools of normal and expanded blastocysts, equally distributed per treatment and per replicate) and bisulfite converted using the EZ methylation direct method (Zymo Research, Freiburg, Germany) following the manufacturer’s guidelines. PCR reactions were carried out using the primers summarized in Table 1. Each PCR reaction contained 16.75 μl H_2_O, 2.5 μl 10x Buffer, 0.5 μl dNTPS (10 mM), 0.5 μl forward and reverse primer (10 μM), 0.25 μl Platinum *Taq* DNA polymerase, 1 μl MgCl_2_ (50 mM) and 3 μl bisulfite DNA template (all products were purchased from Invitrogen Life Technologies). Amplification was as follows: 95 °C for 5 min, then 40x 95 °C for 30 s, variable annealing temperature (see Table 1) for 30 s, 72 °C for 30 s and finally 72 °C for 30 s. Pyrosequencing was carried out on a Pyromark Q24 instrument (Qiagen) as described by Rutledge et al. [8]. Selection of the re-evaluated genes was based on involvement in the most important pathways (mentioned in the results) and that were also present in the top 10 list of differentially methylated genes in IPA to validate changes in DNA methylation of physiologically relevant genes.

**Table 1:** Primers and annealing temperatures used for pyrosequencing

	Forward primer	Reverse primer	Sequencing primer	Annealing temperature
*COL6A3*	AGGGAAGGTTGTTTAGAGGA	ACCTAAAAATACTAATAACTAATCCATCA	GGTTTTATATATAAGTAAAAGAA	57 °C
*APAF1*	TTTGGATGTTTTGTTAGATTGTAGTT	AACTTACCACTACTACCCTCTAC	ATTGGGTTTTGTGTG	54 °C
*RFC4*	TATTTTAAAGGAGATTAGTTTTGGGTGTT	CTCAATTCAATCATAACTCAATCCTATA	ATTGATGTTGAGGTTGAAA	60 °C
*PLSCR3*	TGGTTTTGTTGTTTGGTAGTTAAT	CTCATATAACCAAACCCTTAATCTATC	AGTTTTTTTATTTTAGTAGTTGG	54 °C
*ELOVL1*	GTTTTGTAGAGTTGGTGAGTGTA	ATTTACTATCTAACCAAATCACTTCAC	AGTTGGTGAGTGTATG	59 °C
*TBKBP1*	AGAGATGAGAAGGTTAGAGATTTAAGTAT	AACCCTACCACATCTACATCCTCATA	GTTTTAAATTAGTTGGGAGATA	58 °C
*ERCC1*	TTTTTAGTTTGGATATATAGATATGGGA	ACTCAACCCCACTCATCT	GGATATATAGATATGGGAAATATT	52 °C
*CELF2*	AGGAGAGAGGTGGTAATAATAAAGTTATA	AACCAAAACCCTTTTCTCCA	GTTGGTGATGAATTTGTAG	53 °C
*ZFAND6*	TGGGGAGGAGTATATGTGTATT	CTTATTCTACCATTACTACTATTCTATCTT	ATATGTGTATTTTTTTTTATTATAT	55 °C
*PREX1*	GGTTGGGTATTTATGTTTAGAAATTAAGT	ACCTTCTCTATAATATTTATTCCTCTACC	ATTAAGTGAGAAGGTATTTG	56 °C
*APPL2*	GTAGGTTTTATGGGGAAATTATGTAT	TTCCATACCCTACCTATAAAAAATCTTC	GTTTTTTTTTTTTATTGGGTAGA	55 °C
*GCLC*	GTTAAATGGATGAGGGAGTTTAT	ATTTTACTTCCACTATACCTAACTTT	TGAATTATAGTTTTTTTTTAAAGTG	52 °C

### Statistical analyses

All statistical analyses of developmental competence data were carried out with SPSS 22 (for Windows, Chicago, IL, USA). Cleavage and blastocyst rates were compared between the treatments (BASAL and HIGH COMBI) using a binary logistic regression model. Replicate (random factor), treatment (fixed factor) and their interaction were taken into account. Differences were considered as being significant at *P* < 0.05 and as indicating a trend at 0.05 < *P* < 0.1.

Relative transcript abundance generated through microarray was analysed with FlexArray; statistical data analysis software for gene expression microarrays. Specifically, raw data were corrected by background subtraction, and then normalized within and between each array (Loess and Quantile, respectively). Statistical comparison between treatments was done with the Limma algorithm. A fold change cut-off of 1.5 with *P* < 0.05 was set to identify genes whose expression was significantly differentially regulated. Data were analyzed by Ingenuity Pathways Analysis (IPA; Ingenuity Systems, www.ingenuity.com) software, which served to identify pathways that were differentially expressed between treatments.

Bioinformatic analysis of the EDMA results was performed as described by Shojaei et al. [32]. Briefly, the Limma Bioconductor package was used to apply loess intra-array normalization followed by Quantile inter-array scale normalization to draw the intensities. Normalized intensities were then fitted to a linear model and Bayesian statistics of differential expression were obtained. Probes that exhibited a *P*-value < 0.05 and an absolute log^2^ fold-change of at least 1.5 were considered differentially methylated regions (DMRs). Probes were categorized based on their genomic locations, CpG islands-related characteristics and the type of repeated elements covered. Enrichment ratios were calculated by comparing the proportion of probes in a given category across the whole array to the same proportion within the DMRs. Data were analyzed by Ingenuity Pathways Analysis (IPA; Ingenuity Systems, www.ingenuity.com) software, which served to identify pathways that were differentially methylated between treatments. For interpretation of individual differentially methylated regions, a Benjamini-Hochberg correction was applied to all *P*-values in order to correct for multiple testing, thus reducing the chance of false positives.

Statistical analyses of the pyrosequencing results were performed using the software package R, version 3.1.2. For each gene, several CpG islands were tested in different replicates, which created dependencies between the observations. To account for the dependence between observations within the same CpG island and the same replicate a linear mixed model was fitted, where these dependencies were taken into account by including random effect terms into the model. The treatments (BASAL and HIGH COMBI) were entered as fixed effects. The significance of the fixed effect was tested using an F-test with a Kenward-Roger correction for the degrees of freedom.

## Results

### Experiment 1. Blastocysts originating from NEFA-exposed oocytes: consequences on gene expression and DNA methylation signatures

#### Part 1A. Developmental competence of blastocysts originating from NEFA-exposed oocytes

Cleavage rates of oocytes matured under HIGH COMBI conditions (74.1%) were not significantly different compared to BASAL conditions (72.3%) (*P* > 0.05). However, exposing oocytes to HIGH COMBI conditions resulted in a significant reduction of the proportion of oocytes reaching the blastocyst stage at day 7.5 p.i. (19.3%) compared to BASAL-exposed oocytes (23.2%) (*P* < 0.05). There was also a significantly lower capacity of cleaved zygotes to develop to the blastocyst stage in the HIGH COMBI group (26.0%) compared to the BASAL group (32.1%) (*P* < 0.05). Data on developmental competence are presented in Table 2.

**Table 2:** Developmental competence of bovine oocytes exposed to elevated NEFA concentrations during 24 h of *in vitro* maturation

n (%)	BASAL	HIGH COMBI
Oocytes	990	928
Cleaved	716 (72.3)	688 (74.1)
Blastocysts from oocytes matured	230 (23.2)^a^	179 (19.3)^b^
Blastocysts from cleaved zygotes	230 (32.1)^a^	179 (26.0)^b^

Data indicated with different letters (ab) per row are significantly different (*P* < 0.05)

#### Part 1B. Assessment of DNA methylation patterns using EDMA

**Fig. 1:**
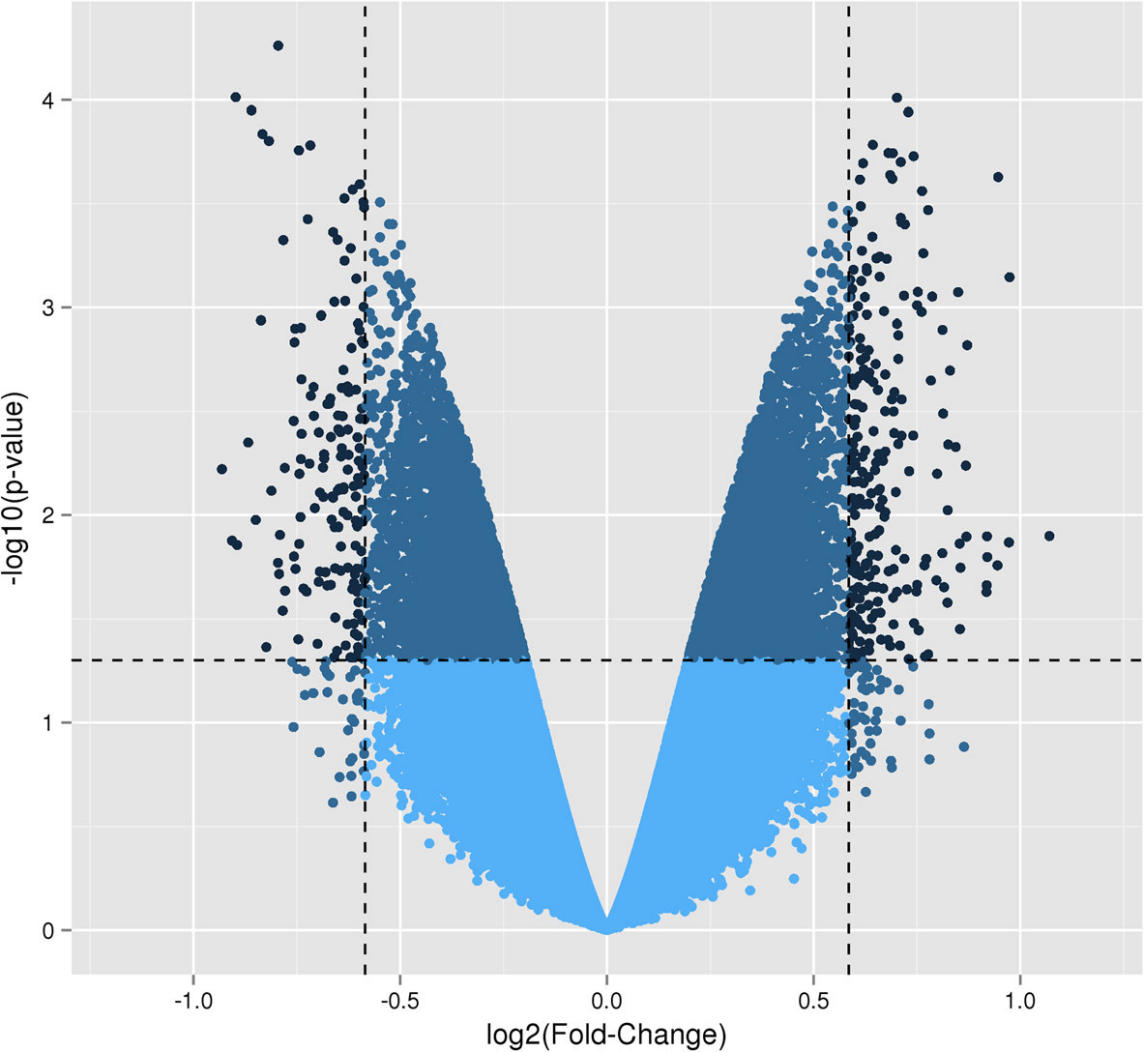
Volcano plot of the DNA methylation microarray results in blastocysts originating from HIGH COMBI-exposed oocytes versus blastocysts originating from BASAL-exposed oocytes. The dots located at the left upper corner are significantly hypomethylated probes (*n* = 155); whereas, the dots located at the right upper corner are significantly hypermethylated probes (*n* = 240) at *P* < 0.05 and fold change >1.5

Comparison of the methylome profile between blastocysts originating from HIGH COMBI-exposed and BASAL-exposed oocytes indicated that 395 loci were differentially methylated (*P* < 0.05 and fold change >1.5). More specifically, 240 loci were hypermethylated in the embryos originating from HIGH COMBI-exposed oocytes as compared to embryos originating from BASAL-exposed oocytes; whereas, DNA methylation at 155 loci was significantly lower in the HIGH COMBI group as compared to the BASAL counterparts as shown in Fig. 1.

**Fig. 2:**
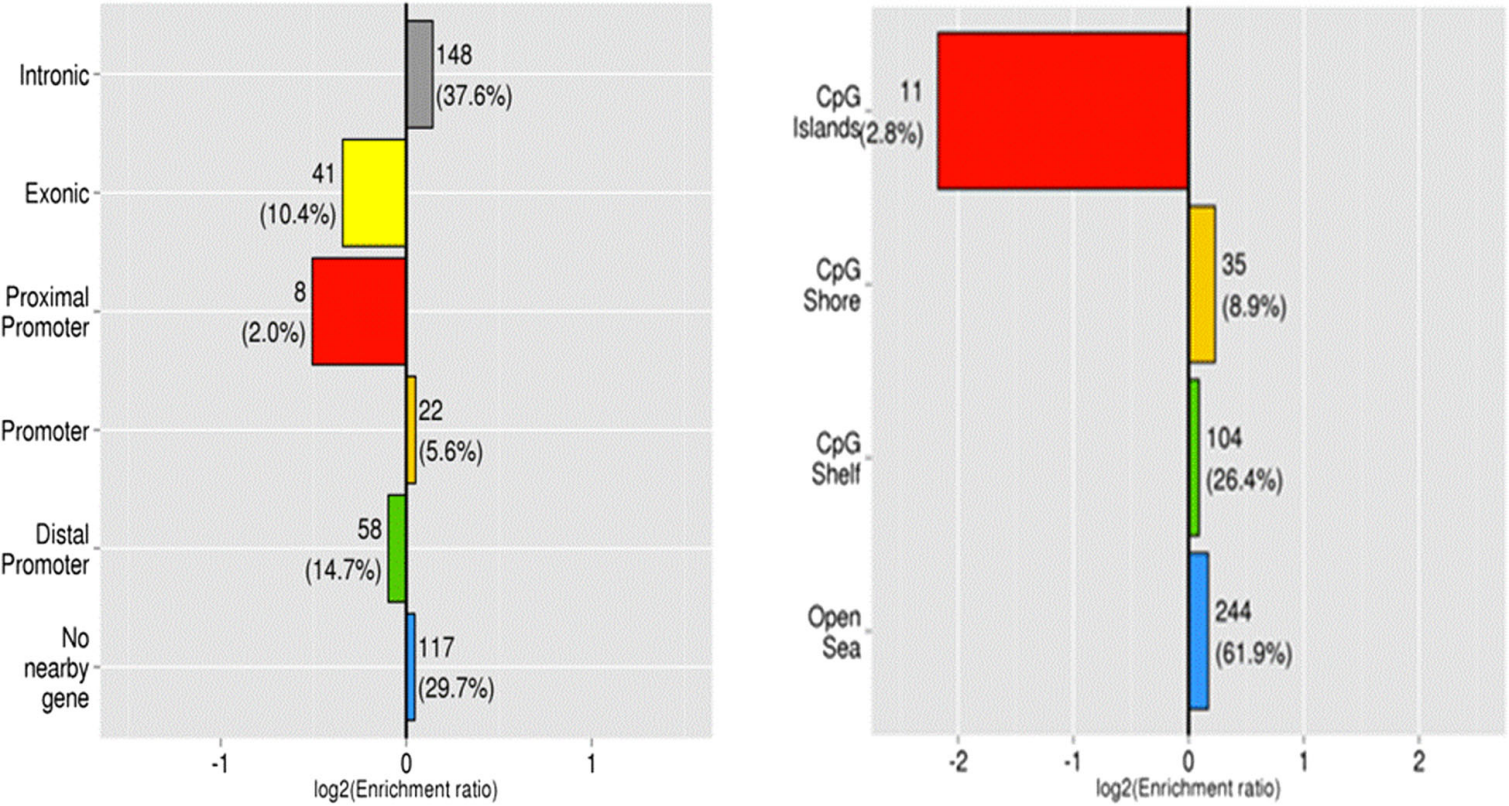
Enrichment of differentially methylated regions (DMRs) according to genic regions (*left*) and distance from CpG island (*right*) (with a CpG shore, CpG shelve and open sea located 1–2 kbp, 2–4 kbp and >4 kbp away from the nearest CpG island, respectively) in blastocysts originating from HIGH COMBI- exposed oocytes. Enrichment is defined as log2 (proportion of DMR probes within category / proportion of total probes in that category). A bar of length 1 indicates that there are twice as many differentially methylated probes in that category as expected. The numbers besides a bar represent how many DMRs fit in that category and the percentage of all DMRs in that category

Enrichment analysis of the data also identified which specific genomic features (genomic localisation and distance to CpG islands) were present in blastocysts derived from HIGH COMBI-exposed oocytes and this is presented in Fig. 2. The proximal promoter, promoter and distal promoter regions were defined as the first 1kbp, 5kbp and 50kbp of the transcription start site. There appeared to be a tendency to conserve DNA methylation within proximal promoters, exonic regions and distal promoters. Conversely, differential DNA methylation was more likely to occur at intronic and intergenic regions and promoter sites. In addition, analysis of the distance of probes relative to CpG islands was performed. The probes were categorized into CpG shores, CpG shelfs and open seas when they were located 1-2 kbp, 2-4 kbp or >4 kbp away from the nearest CpG island, respectively. Differentially methylated regions were found in CpG shores, CpG shelfs and open seas in the HIGH COMBI group. DNA Methylation at CpG islands tended to be more stable and was not observed to be affected by high concentrations of NEFAs during oocyte maturation.

**Fig. 3:**
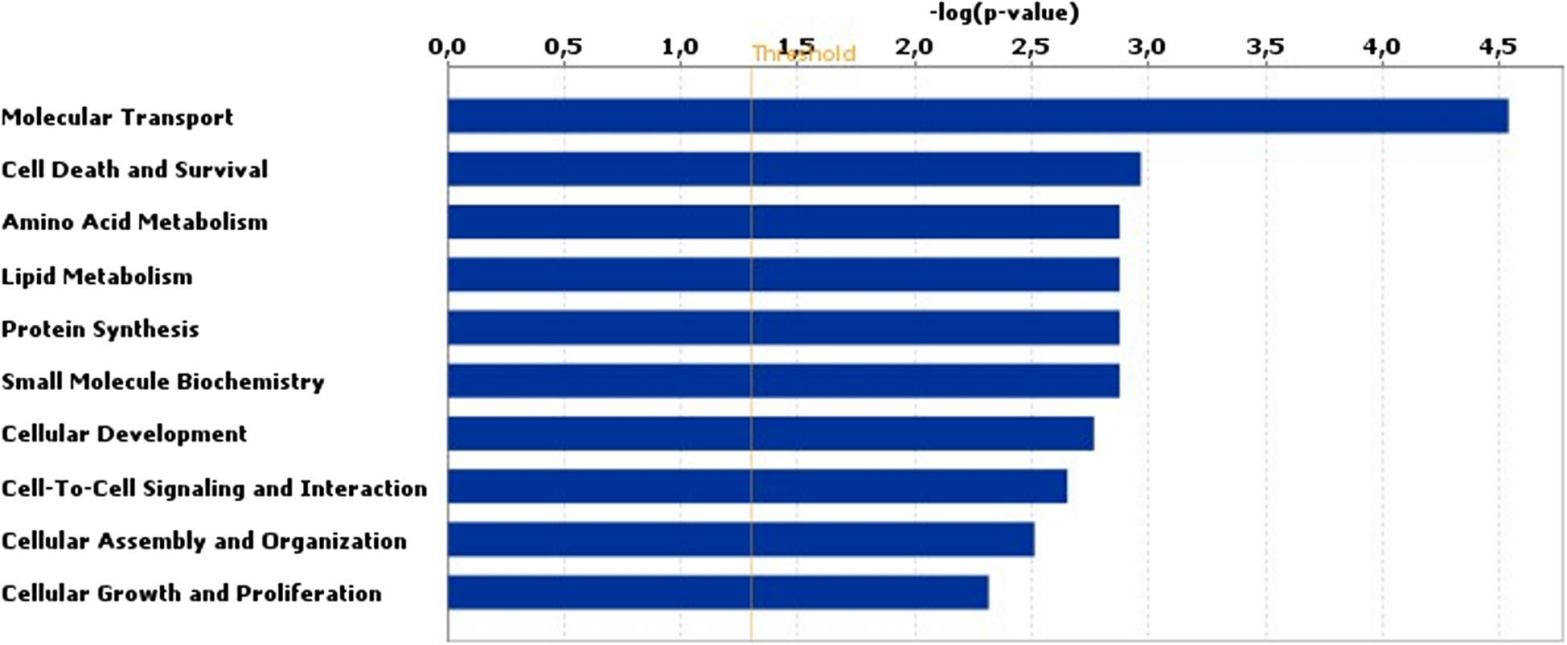
Results of biofunction analysis with the Ingenuity software for a DNA methylation microarray. The top 10 pathways affected in blastocysts originating from HIGH COMBI-exposed oocytes versus blastocysts originating from BASAL-exposed oocytes are arranged by descending *P*-value

Among the loci that were differentially methylated between embryos from HIGH COMBI-treated and BASAL-treated oocytes, 148 hypermethylated and 120 hypomethylated regions mapped to coding-regions and were used for further analysis using IPA. Output from IPA analyses revealed that these coding-regions were associated with cellular development and function, metabolism, cell cycle, cell survival and death and gene expression (see Fig. 3). When applying a Benjamini-Hochberg correction, no differentially methylated regions displayed an adjusted *P*-value < 0.05 when comparing treatments.

The DNA methylation patterns were integrated with the transcriptome data reported by Van Hoeck et al. [5] to determine possible associations. In order to obtain a general overview of pathways affected on transcriptome and methylome level, microarray data without FDR correction were used for IPA analysis. In summary, transcriptomic comparison between blastocysts originating from oocytes matured under HIGH COMBI conditions compared to BASAL conditions revealed that 190 genes were differently expressed of which 85 up- and 105 down-regulated genes. These data were validated using qRT-PCR. The correlation between specific differentially methylated loci and expressed genes is presented in Table 3.

**Table 3:** Relation between differentially methylated genes and downstream expressed genes of blastocysts originating from oocytes exposed to high NEFA concentrations

Associated pathways	Differentially methylated genes	Downstream differentially expressed genes
Apoptosis	*TP53*	*SIRT1, MAD2L1, FAM3C, CDC7, CD47, HERC5, HSPD1,SCP2, UBL3, ID3*
Embryo implantation	*LIF*	*GPCPD1, CYP11A1*
Gene transcription	*CTBP1*	*SIRT 1*
	*IGF1R*	*PDCD10, SCP2, KRT19, CYP11A1*
Immune response	*TCR*	*HERC5, HSPD1, GBP4, ID3*
	*RXRB*	*CCL17*
Metabolism	*G6PC*	*CYP11A1*
	*LEP*	*SC4MOL, SCP2, TIMP1*
	*PEPCK*	*CYP11A1*

#### Part 1C. Targeted analysis of DNA methylation

**Fig. 4:**
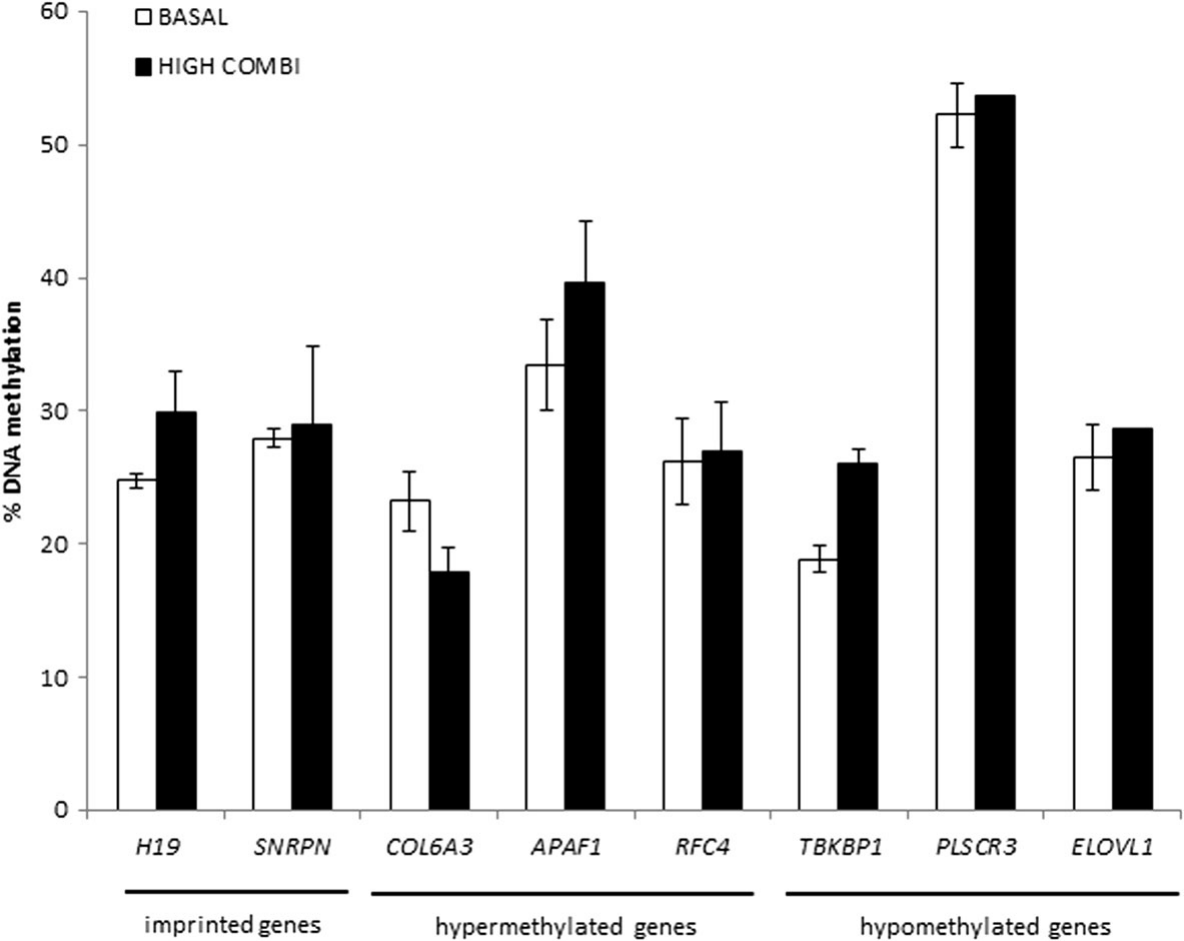
Methylation patterns of *H19*, *SNRPN*, *COL6A3*, *APAF1*, *RFC4*, *PLSCR3*, *ELOVL1* and *TBKBP1* in blastocysts originating from HIGH COMBI-exposed oocytes versus blastocysts originating from BASAL-exposed oocytes. Bars are presented as means ± SEM

Methylation profiles of specific genes of interest were evaluated using pyrosequencing techniques in embryos from HIGH COMBI-treated and BASAL-treated oocytes. The selected genes (*COL6A3*, *APAF1*, *RFC4*, *PLSCR3*, *ELOVL1* and *TBKBP1)* are associated with pathways such as apoptosis, metabolism, gene transcription and inflammatory response. In addition, DNA methylation at two imprinted genes, *H19* and *SNRPN*, was also determined in embryos derived from HGH COMBI- and BASAL-treated oocytes. The degree of DNA methylation of these imprinted genes is known and therefore, they were implemented as controls (see Fig. 4).

Methylation at the two imprinted genes (*SNRPN* and *H19*) was not significantly different between embryos from BASAL- and HIGH COMBI-exposed oocytes (28.0% compared to 29.0% for *SNRPN* and 24.8% compared to 29.9% for *H19*). Only *APAF1* (*P* = 0.40) and, to a lesser extent, *RFC4* (*P* = 0.20) showed hypermethylation (not statistically significant) in embryos from HIGH COMBI-treated as compared to embryos from BASAL-treated oocytes as was observed in the microarray results, while none of the hypomethylated genes (based on the micro-array results) could be validated using pyrosequencing.

### Experiment 2. Blastocysts originating from NEFA-exposed embryos: consequences on gene expression and DNA methylation signatures

#### Part 2A. Developmental competence of blastocysts originating from NEFA-exposed embryos

Cleavage rates of embryos cultured under HIGH COMBI conditions (65.2%) were significantly different compared to BASAL conditions (75.6%) (*P* < 0.05). Moreover, exposing embryos to HIGH COMBI conditions resulted in a significant reduction of the proportion of embryos reaching the blastocyst stage at day 7.5 p.i. (18.2%) compared to BASAL-exposed oocytes (25.3%) (*P* < 0.05). There was also a significantly lower capacity of cleaved zygotes to develop to the blastocyst stage in the HIGH COMBI group (27.9%) compared to the BASAL group (33.5%) (*P* < 0.05). Data on developmental competence are presented in Table 4.

**Table 4:** Developmental competence of bovine embryos exposed to elevated NEFA concentrations during 6.5 days of in vitro culture

n (%)	BASAL	HIGH COMBI
Oocytes	1217	1199
Cleaved	920 (75.6)^a^	782 (65.2)^b^
Blastocysts from oocytes matured	308 (25.3)^a^	218 (18.2)^b^
Blastocysts from cleaved zygotes	308 (33.5)^a^	218 (27.9)^b^

Data indicated with different letters (ab) per row are significantly different (*P* < 0.05)

#### Part 2B. Consequences for expression patterns of genes involved in epigenetic pathways using microarray approach

**Fig. 5:**
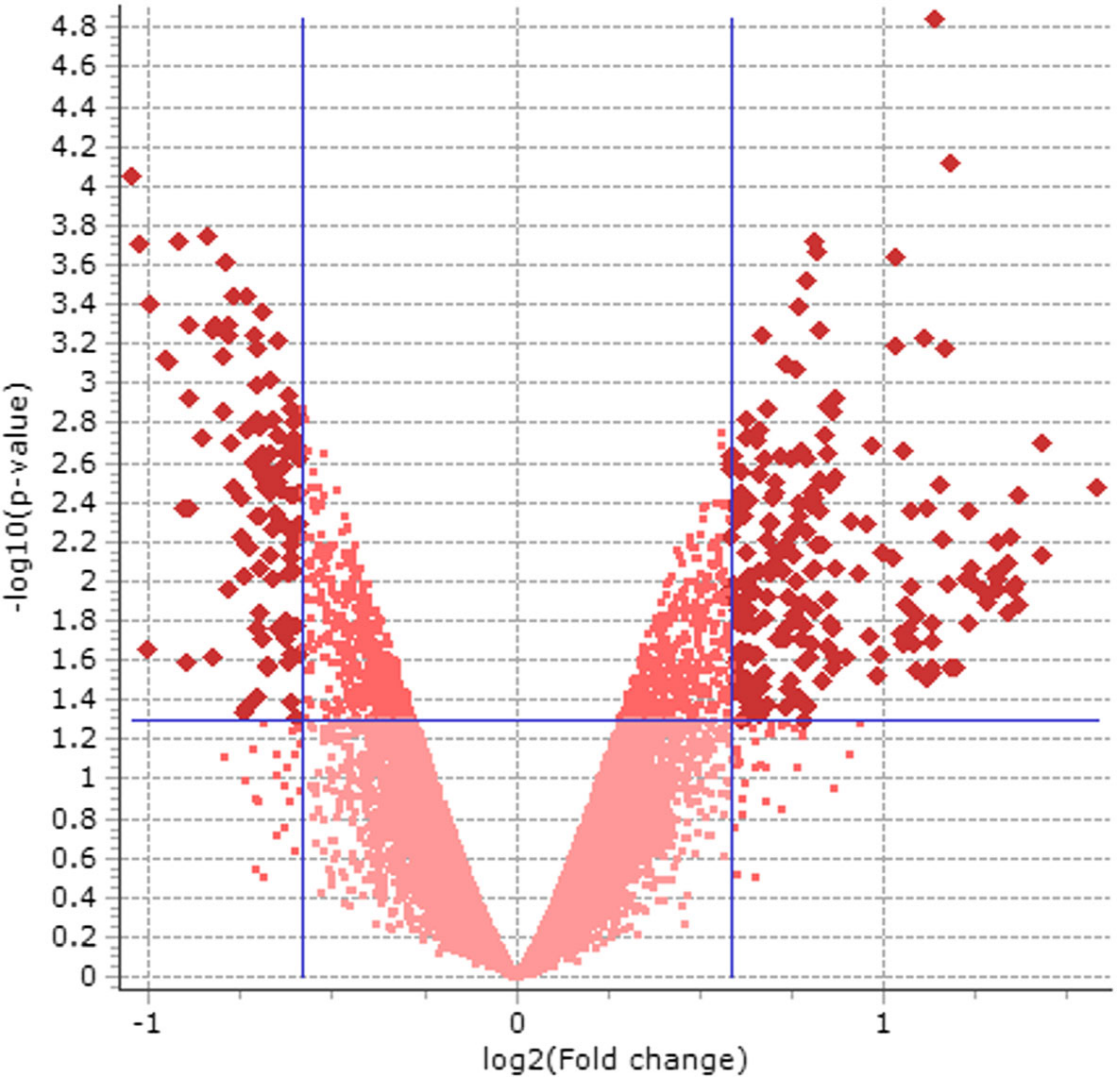
Volcano plot of the microarray results in blastocysts originating from HIGH COMBI-exposed embryos versus blastocysts originating from BASAL-exposed embryos. The dots which are located in the left upper corner of the plot are the significantly down-regulated genes; whereas, the dots located in the right upper corner of the plot are those which are significantly up-regulated (*P* < 0.05 and fold change >1.5)

The microarray platform identified 311 differentially expressed genes (*P* < 0.05 and fold change > 1.5) between blastocysts originating from embryos cultured under HIGH COMBI and BASAL conditions. More specifically, 206 genes were up-regulated; whereas, 105 genes were down-regulated (see Fig. 5). To validate the results of the microarray with an independent analysis, we used qRT-PCR (as described by Van Hoeck et al. [5]) to examine a subset of 5 genes from the differentially regulated gene list. The pattern of 4 of the transcripts mirrored that seen on the arrays in terms of the direction of the change (up- or down-regulated).

**Fig. 6:**
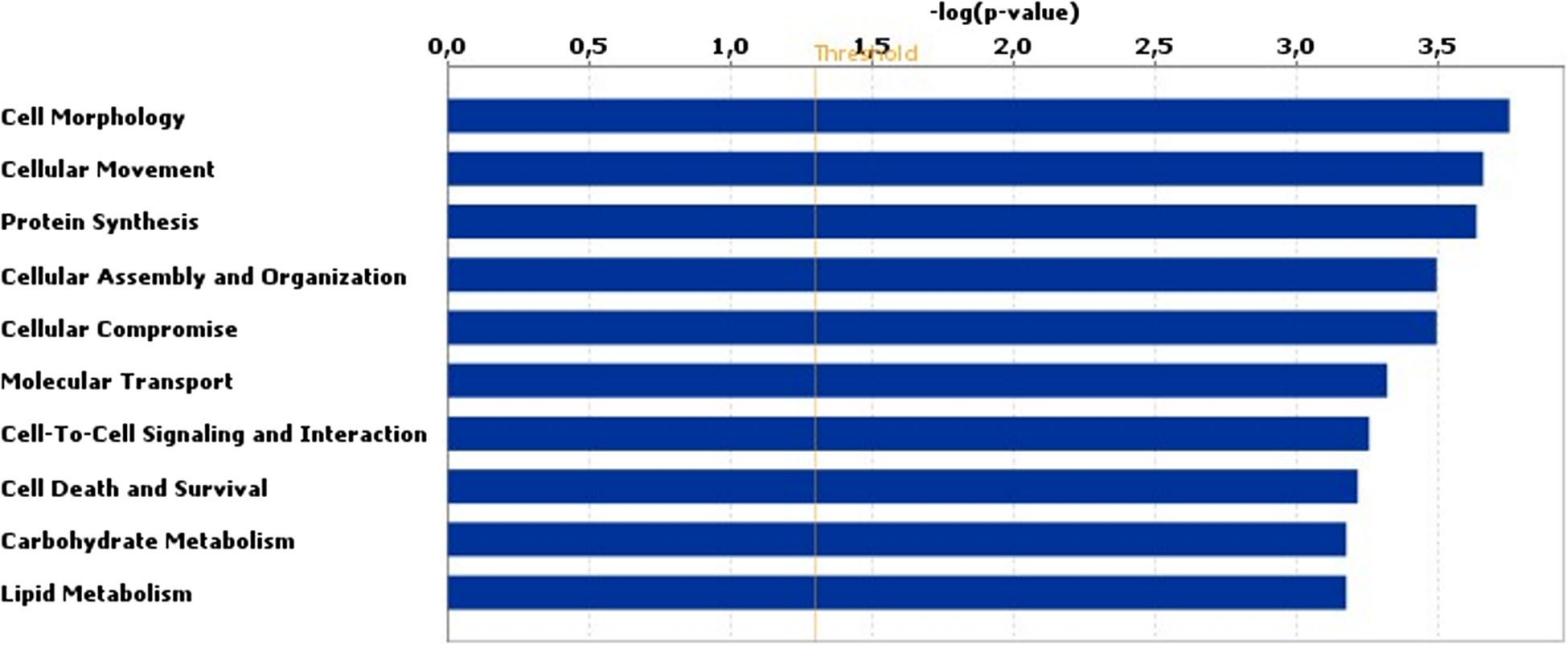
Results of biofunction analysis with the Ingenuity software for microarray. The top 10 pathways affected in blastocysts originating from HIGH COMBI-exposed embryos versus blastocysts originating from BASAL-exposed embryos are arranged by descending *P*-value

IPA analysis indicated that the following networks were affected by the HIGH COMBI treatment during embryo culture; cell morphology, cell-to-cell signaling and interaction, hematological system development and function, lipid metabolism, small molecule biochemistry, vitamin and mineral metabolism, lipid metabolism and molecular transport (see Fig. 6).

Based on the transcriptomic patterns of blastocysts originating from HIGH COMBI-treated embryos relative to blastocysts originating from BASAL-treated embryos, changes in only a single gene associated with epigenetic modifications was identified, *SHMT1. SHMT1* was down-regulated in blastocysts that were cultured under HIGH COMBI conditions (*P* < 0.001 and fold change = −1.892).

#### Part 2C. Assessment of DNA methylation patterns using EDMA

**Fig. 7:**
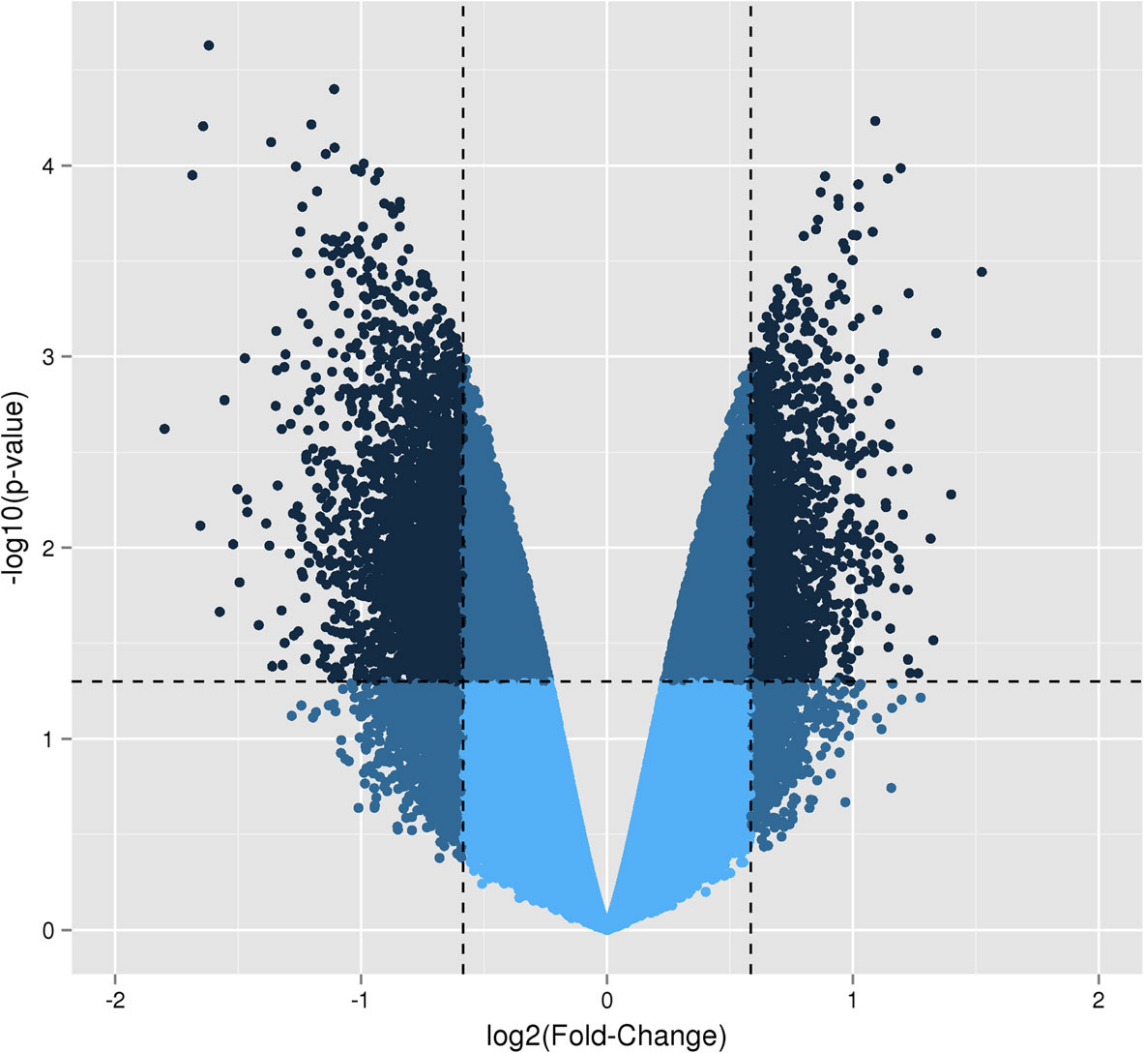
Volcano plot of the microarray results in blastocysts originating from HIGH COMBI-exposed embryos versus blastocysts originating from BASAL-exposed embryos. The dots located in the left upper corner are statistically significantly hypomethylated probes (*n* = 879); whereas, the dots located in the right upper corner are statistically significantly hypermethylated probes (*n* = 697) (*P* < 0.05 and fold change >1.5)

Comparison of the methylome profile between blastocysts, originating from embryos cultured under HIGH COMBI and those cultured under BASAL conditions, indicated that 4,671 loci were differentially methylated (*P* < 0.05 and fold change >1.5). Results are summarised in Fig. 7. More specifically, 1,912 loci were hypermethylated in HIGH COMBI as compared to BASAL embryos; whereas, DNA methylation of 2,759 loci was significantly lower in HIGH COMBI as compared to BASAL treated embryos.

**Fig. 8:**
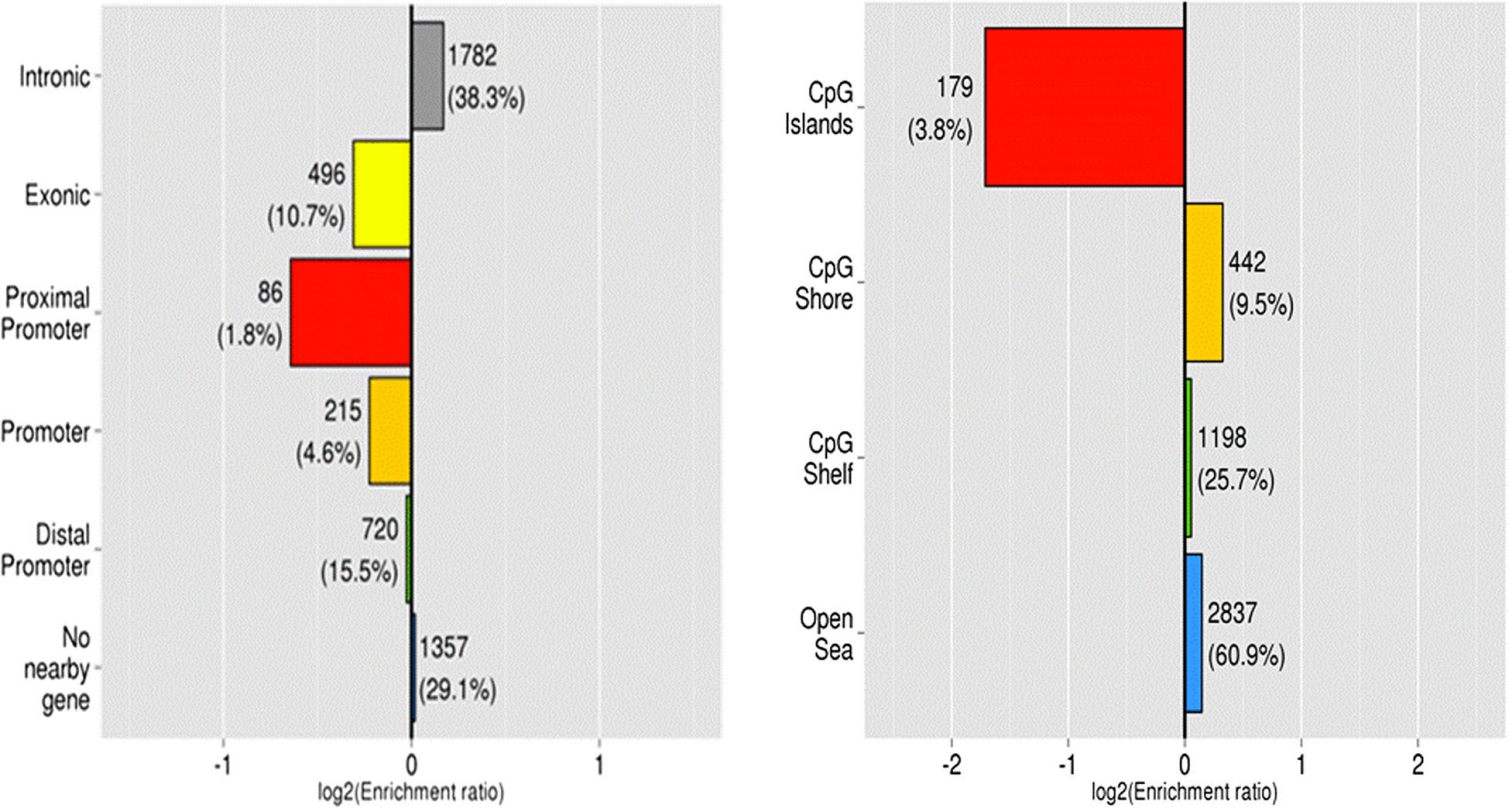
Enrichment of differentially methylated regions (DMRs) according to genic regions (*left*) and distance from CpG island (*right*) (with a CpG shore, CpG shelve and open sea located 1–2 kbp, 2–4 kbp and >4 kbp away from the nearest CpG island, respectively) in blastocysts originating from HIGH COMBI-exposed embryos. Enrichment is defined as log2 (proportion of DMR probes within category / proportion of total probes in that category). A bar of length 1 indicates that there are twice as many differentially methylated probes in that category as expected. The numbers besides a bar represent how many DMRs fit in that category and the percentage of all DMRs in that category

Enrichment analysis of the data also determined which genomic features were present following exposure of the developing embryo for HIGH COMBI conditions and this is presented in Fig. 8. There was again a tendency to changes in methylation at intronic or intergenic regions. The level of methylation at promoter sites and exonic regions was not affected by the HIGH COMBI treatment. The distance of DMRs to CpG islands was determined as described above. DMRs were found in CpG shores, CpG shelves and open seas. Methylation at CpG islands tended to be conserved.

**Fig. 9:**
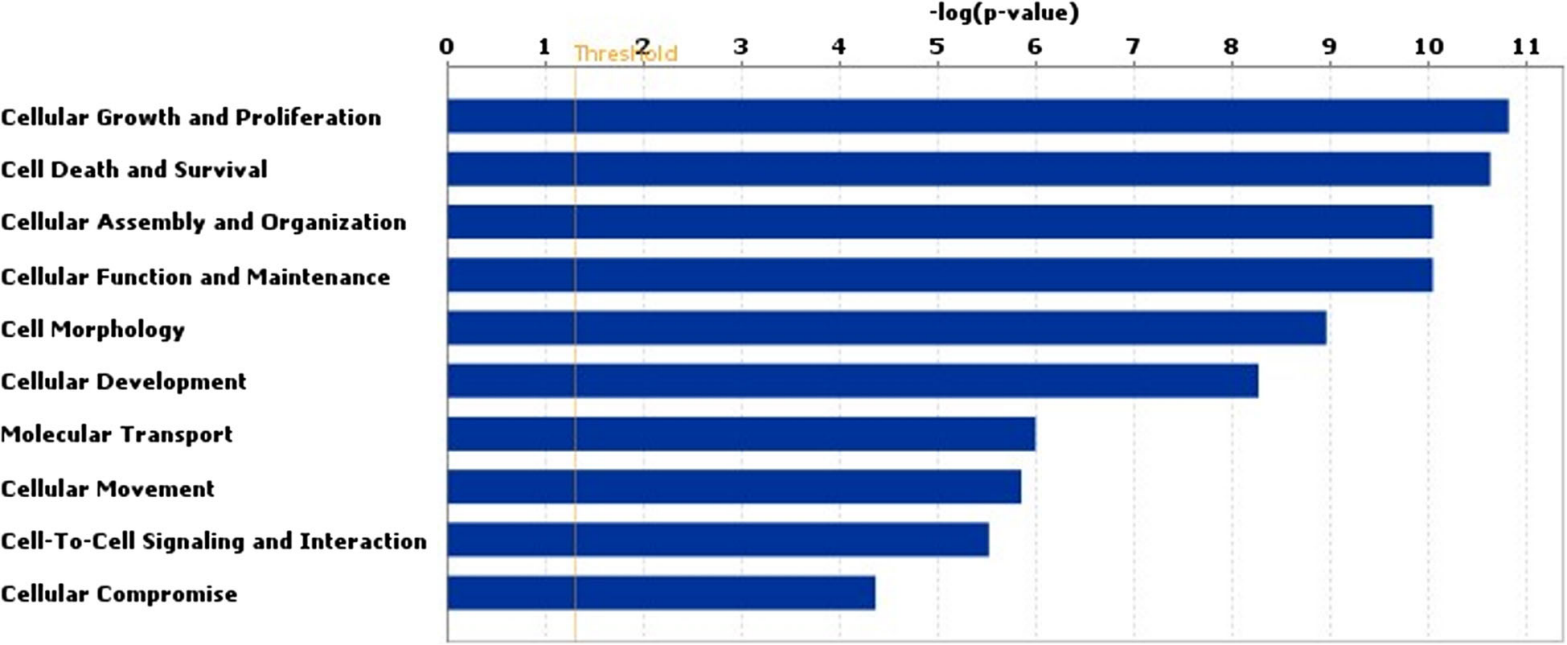
Results of biofunction analysis with the Ingenuity software for a DNA methylation microarray. The top 10 pathways affected in blastocysts originating from HIGH COMBI-exposed embryos versus blastocysts originating from BASAL-exposed embryos are arranged by descending *P*-value

Of the loci that were differentially methylated between HIGH COMBI and BASAL embryos, 1576 mapped to coding-regions and were used for subsequent pathway analysis. Of these, 697 were hypermethylated and 879 were hypomethylated. Using these genes as input for IPA it was determined that the main molecular and cellular functions altered were overall related to cellular development and function, metabolism, cell cycle, cell death and survival and gene expression (see Fig. 9). When applying a Benjamini-Hochberg correction, no differentially methylated regions had an adjusted *P*-value < 0.05 when comparing treatments.

The DNA methylation data were compared with transcriptome profiles to investigate a possible association between changes in DNA methylation and gene expression. In order to maximize visualization of similar affected pathways, microarray data before FDR correction were applied for IPA analysis. The latter information is not used to draw conclusions at individual gene level. The correlation between specific differentially methylated loci and expressed genes is presented in Table 5.

**Table 5:** Relation between differentially methylated genes and downstream expressed genes of blastocysts originating from embryos exposed to high NEFA concentrations

Associated pathways	Differentially methylated genes	Downstream differentially expressed genes
Apoptosis	*TP53*	*SERPINE1, DCTN2, CNN3, BHLHB2, MTDH, ASNS, GLB1, CNN1, HMGCS1, CCND2, LBR, ANKH, NANOG, SEC61B, HSPG2, COX7A2, GZMB, IGFBP7*
Autophagy	*ATG16L1*	*UPK1A*
Cell cycle	*TERF2*	*GLB1*
Cell differentiation	*FGFR2*	*CCND2, NANOG, NR5A2*
Cell interaction	Alpha catenin	*RHOC, IGFBP7, TWIST2*
Cell signaling	*ERK1/2*	*SERPINE1, CCND2, LDLR, NANOG, RHOC*
	*ITGAV*	*SERPINE1, LDLR*
Cell survival	*FGF10*	*SCD, LDLR, LFNG*
Cellular development	*THPO*	*CCND2, GP1BA*
Hormonal regulation	estrogen receptor	*SERPINE1, CCND2, LDLR, MUC1*
	*FSH*	*SERPINE1, CCND2, LDLR, AQP3, ARL4C, GZMB, CARD10, DUSP14, GATA4*
	*FSHR*	*GATA4, GZMB∆*
Immune response	*CD28*	*TOM1L1, CCND2, TXNRD1, PA2G4, AQP3, SEC61B, RHOC, GZMB, DUSP14*
	*Cg*	*BHLHB2, CCND2, LDLR, S100A10, NR5A2, GATA4*
	*TCR*	*LBR, PA2G4, GZMB, ARPP21, LMNB1*
Ion transport	*WNK1*	*SERPINE1*
Gene transcription	*Creb*	*MIF, HSPB8, LDLR, ELL*
	*PRDM16*	*SERPINE1*
	*SMARCA4*	*SERPINE1, MUC1, ASNS, FLNB, TXNRD1, ARL4C*

#### Part 2D. Targeted analysis of DNA methylation

**Fig. 10:**
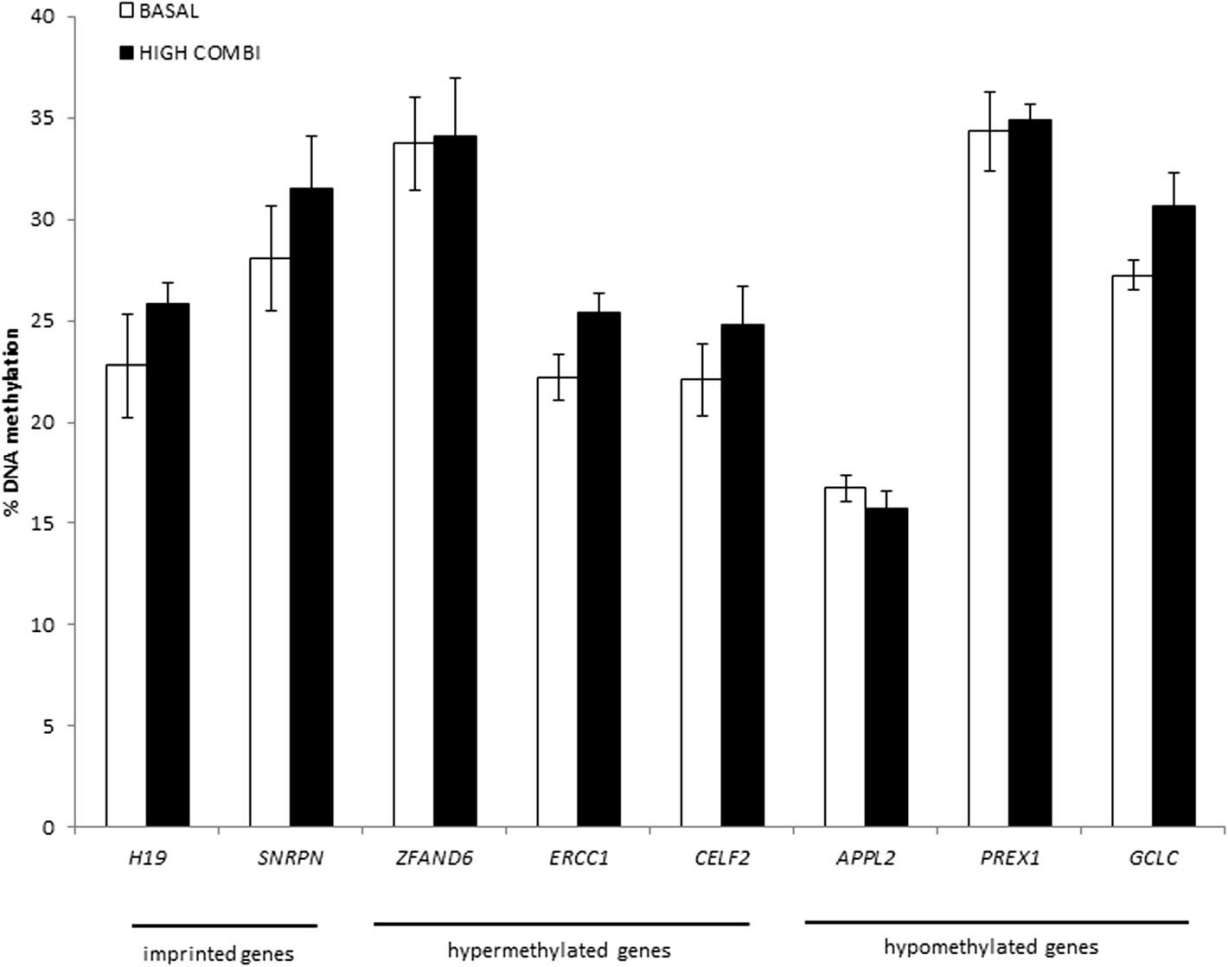
Methylation patterns of *ERCC1*, *CELF2*, *ZFAND6*, *PREX1*, *APPL2* and *GCLC* in blastocysts originating from HIGH COMBI-exposed embryos versus blastocysts originating from BASAL-exposed embryos. Bars are presented as means ± SEM

In order to re-evaluate the DNA methylation microarray results, we investigated the methylation profiles of *ERCC1*, *CELF2*, *ZFAND6*, *PREX1*, *APPL2* and *GCLC* in HIGH COMBI and BASAL embryos using pyrosequencing. The average methylation level at two imprinted gene DMRs, *H19* and *SNRPN*, was also determined in both BASAL and HIGH COMBI embryos as controls. The average methylation level of the selected genes of HIGH COMBI- compared to BASAL-treated embryos is presented in Fig. 10.

*SNRPN* had an average methylation level of 28.1% in BASAL embryos compared to 31.5% in HIGH COMBI embryos. For the H19 DMR, similar methylation levels were observed in BASAL (22.8%) and HIGH COMBI (25.8%) embryos. *ERCC1* (*P* = 0.16), *CELF2* (*P* = 0.52) and *ZFAND6* (*P* = 0.81) showed hypermethylation in HIGH COMBI embryos compared to control embryos as was observed in the microarray results (not statistically significant). Hypomethylation of HIGH COMBI embryos compared to BASAL embryos was also observed for *APPL2* (*P* = 0.45) (not statistically significant).

## Discussion

It has been shown previously that elevated NEFA concentrations, as present in several metabolic disorders, can impair oocyte and embryo developmental competence and influence transcriptomic and phenotypic fingerprints of resultant blastocysts [5, 7]. Here, we provide evidence that alterations to the epigenome occur in response to conditions of elevated NEFA concentrations during oocyte maturation or early embryo culture. Major affected pathways are related to cell death and survival, immunity, metabolism and metabolic disorders. Although interpretation of methylation rates at individual loci remained difficult as high rates of variation were observed in methylation patterns between blastocysts within treatment groups, the data shed a clear light on the heatmap of pathways affected by an adverse environment. These epigenetic changes may contribute to the observed transcriptomic and phenotypic alterations and could persist during further development.

Following exposure of oocytes to pathophysiological concentrations of NEFAs, the main pathways affected at the transcriptomic level in blastocysts were related to lipid and carbohydrate metabolism, small molecule biochemistry and cellular development as described by Van Hoeck et al. [5]. Furthermore, transcriptomic data-analysis revealed that NEFA exposure during oocyte maturation affected the expression of genes related to epigenetic programming; *HIST1H2BN*, *UBA2* and *PPP1CB*. For example, *HIST1H2BN* encodes a histone that is a member of the histone H2B family and functions in the compaction of chromatin and thus in histone modifications [33]. Such histone modifications in fetal primates have been previously linked to conditions of maternal overnutrition [34]. Therefore, we assumed that high NEFA concentrations, associated with maternal metabolic disorders, also impact on epigenetic mechanisms.

Genome-wide analysis revealed that elevated NEFAs during oocyte maturation influenced overall DNA methylation patterns in the resultant blastocysts, affecting many DMRs associated with cell death and survival and cellular metabolism. Aberrant DNA methylation patterns were observed in pathways related to caspase activation, p53-induced apoptosis, Ras-signalling and Wnt-signalling. The latter observations are in line with the phenotypic data published by Van Hoeck et al. [4], reporting an increased apoptotic cell ratio in blastocysts from HIGH COMBI- as compared to BASAL-exposed oocytes. Previously, Ge et al. [35] demonstrated an association between maternal metabolic disorders and epigenetic regulation of oocyte metabolism in mice. Similarly, our data revealed methylation changes in lipid transfer and synthesis pathways in blastocysts originating from HIGH COMBI-exposed oocytes. Similar transcriptome and phenotypic fingerprints of blastocysts originating from HIGH COMBI-exposed oocytes were previously described [5, 7]. Epigenetic alterations were also observed in pathological processes such as obesity and insulin-dependent diabetes. These alterations in insulin signalling are interesting since an excessive uptake and accumulation of fatty acids in somatic cell lines is known to be closely associated with type II-diabetes [36], as for example shown in muscle cells by Bilan et al. [37]. Furthermore, Van Hoeck et al. [4, 5] observed phenotypic features associated with glucose intolerance in blastocysts originating from HIGH COMBI-exposed oocytes.

Following exposure of developing embryos to elevated NEFA concentrations during culture, the main pathways affected in resultant blastocysts using transcriptomic analysis were similar to those in the IVM experiment, i.e. lipid and carbohydrate metabolism, small molecule biochemistry and cellular development. Down-regulation of genes related to embryonic cell growth, cell differentiation and cell-cell interaction suggests a reduced developmental competence of blastocysts from HIGH COMBI-treated embryos. Interestingly, HIGH COMBI exposure during embryo culture also affected the expression of a gene required for DNA synthesis and methylation, *SHMT1*. Down-regulation of *SHMT1* has previously been shown to increase methionine formation and thus might affect DNA methylation [38]. Overall changes in DNA methylation were indeed observed in blastocysts from HIGH COMBI-exposed embryos.

DNA methylation analysis of blastocysts originating from HIGH COMBI-exposed embryos revealed altered methylation at loci of genes related to pathways associated with apoptosis, such as the Ras/MAPK signalling [39], the formation of antioxidants [40] and mitochondrial dysfunction [41]. ‘Molecular transport’ pathways, for example involved in regulation of endosomal cholesterol trafficking by recycling sterol carrier proteins, were significantly affected [42]. Overall lipid metabolism was affected by high NEFA exposure with particular focus on pathways as adipogenesis, adipocyte differentiation and fatty acid synthesis [43]. Exposure of embryos to high NEFA concentrations not only influenced lipid but also glucose metabolism, as for example observed in glucose transport [44–46]. Some metabolism- or immune response-related genes are also associated with obesity and insulin resistance [41, 47–50]. Alterations in these pathways may further affect the offspring since maternal metabolic disorders have been shown to increase the risk of developing metabolic disorders in the offspring [51].

Even though the aim of the present study was to achieve a broad overview of affected pathways, an attempt was made to verify DNA methylation at gene level in both IVM and IVC experiments by three different approaches.

Validation of EDMA array data using other techniques has been proven difficult due to the non-linear amplification steps involved in array generation. Shojaei et al. [32] have shown that pyrosequencing offers a partial solution to this problem, whereby methylation trends (gain or loss) generally replicate. Therefore, we chose this technique in the first approach to validate the array. Only two and four of the six genes showed a similar trend in methylation direction as observed with the microarray in the IVM and IVC experiment, respectively. None of these differences were statistically significant due to the high variability and thus pyrosequencing could not confirm the microarray results. In this context, such a high level of variation in average methylation between replicates in bovine day 7 blastocysts has also been observed by O’Doherty and McGettigan [12]. The latter authors suggested that the observed variability in DNA methylation at day 7 may be due to preferential amplification of maternal or paternal alleles, overrepresentation of DNA from the ICM or the TE or differences between gender. This variation in DNA methylation between blastocysts was previously described by Dobbs et al. [17].

In the second approach, gene-specific methylation was assessed by applying an FDR correction to the microarray data. The digestion protocol targets specific cytosines that are not necessarily alone in the methylation landscape creating a bias towards the analysis of these sites compared to all sites [32]. In the context of relatively small individual changes for any specific DMR, the power obtained is not sufficient to create a list of defined targets. Therefore, a Benjamini-Hochberg correction was applied to the list of DMRs to adjust for the number of targets. No DMRs remained when the stringency was raised to the maximum, indicating that the differences due to the NEFA treatment were minimal at gene level. Additionally, this test is highly sensitive to the number of biological replicates [52] and the present study used, due to practical limitations, four biological replicates. Bioinformatic tools, such as IPA, are consequently required to identify if the distribution of the hundreds of DMRs is random or associated with specific functions of pathways. Analysis of the DMR lists with the IPA software unraveled significant changes in multiple pathways due to NEFA exposure as explained above.

In the last approach, methylome data were compared with transcriptome data to examine if genes displayed changes in both DNA methylation and gene expression signatures. It is generally accepted that methylation is implied in expressing/silencing of the gene [53]. However, no differentially methylated loci did result in modified gene expression patterns. This discrepancy between methylation and expression was also observed by Salilew-Wondim et al. [54] who suggested that changes in DNA methylation could not only result in modifications of mRNA expression but also alter the expression of other non-coding RNAs. Moreover, epigenetic regulation of gene expression is complex and also involves additional epigenetic mechanisms like histone modifications [33]. Besides the discrepancy of transcriptome and methylome changes of individual genes, comparable changes were observed at the level of pathways. In this context, IPA analysis confirmed an overlap between pathways displaying altered gene expression and DNA methylation and thus suggests that DNA methylation is an epigenetic trigger for changes in gene expression due to elevated NEFA exposure.

Based on the lack of validation using these different approaches, we conclude that in the present study the EDMA array was not able to detect changes in DNA methylation in order to draw specific conclusions at the level of an individual gene. Aside from the methodological restrictions, the blastocyst as such is a difficult target for methylation analysis. Rekik et al. [55] observed very dynamic gene expression profiles during a relatively short time period encompassing blastocyst formation and hatching. Similarly, changes in DNA methylation fingerprints emerge from fertilization onwards. DNA methylation declines as the embryo undergoes successive cell divisions to a nadir at the 6–8 cell stage and increases thereafter. At blastocyst level, DNA methylation varies depending upon gender and cell lineage as demonstrated by Dobbs et al. [17]. Even imprinted genes, which are resistant to epigenetic reprogramming, may be susceptible to instability at the blastocyst stage [56]. We tried to minimize these effects by pooling embryos of similar stages across treatments and replicates to generate comparable biological replicates although the extent of DNA methylation still remains naturally variable among samples of similar origin. Notwithstanding these limitations, the blastocyst is an interesting target to investigate. Transcriptome studies have extensively shown how early embryos interact with their immediate microenvironment [31, 57, 58]. Epigenetic reprogramming at this stage puts the embryo at risk of programming errors with potential long-term consequences due to an adverse environment [23].

**Fig. 11:**
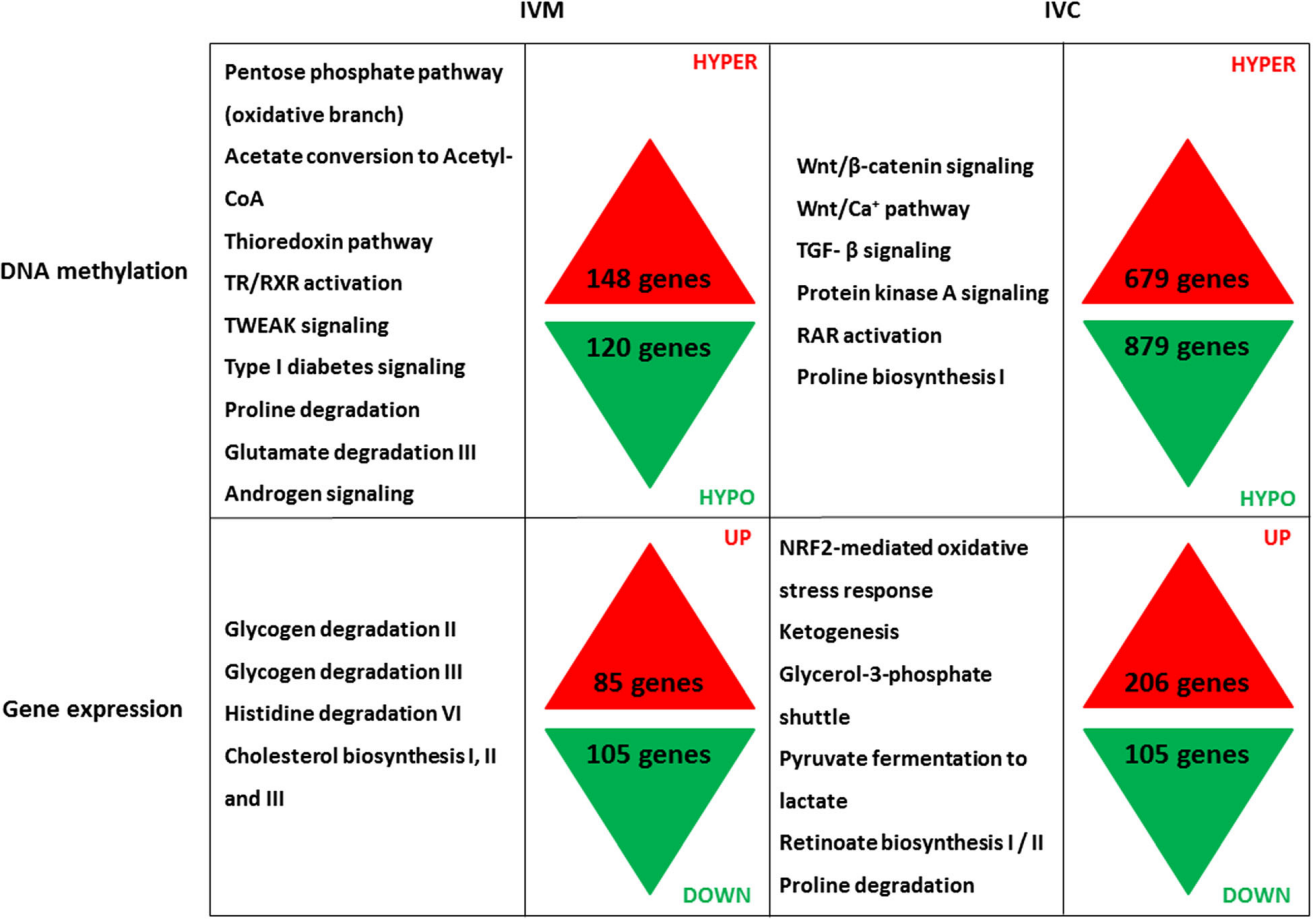
Main canonical pathways showing significant differences in DNA methylation (*upper panel*) and gene expression (*lower panel*) in the IVM (*left table*) and the IVC (*right table*) experiment. Number of genes showing hyper- (*red*) and hypo- (*green*) methylation (*upper panel*) or up- (*red*) and down- (*green*) regulation (*lower panel*) in blastocysts originating from HIGH COMBI-exposed oocytes versus blastocysts originating from BASAL-exposed oocytes (IVM) and in blastocysts originating from HIGH COMBI-exposed embryos versus blastocysts originating from BASAL-exposed embryos (IVC)

Comparison of data from NEFA exposure during IVM (24 h) and IVC (168 h) demonstrated that a higher number of differentially expressed genes and aberrantly methylated loci were present in response to elevated NEFA concentrations during IVC compared to IVM. All data regarding changes in DNA methylation, gene expression profile and associated affected canonical pathways in the IVM and IVC experiment are summarized in Fig. 11. Indeed, it is likely that oocytes are less sensitive to NEFA exposure than embryos due to the presence of the surrounding cumulus cells. It has been observed that the cumulus cells incorporate fatty acids as lipids and thereby protect the oocyte from in vitro induced lipotoxic effects [59, 60]. Moreover, oocytes rely on oxidative fatty acid metabolism during final maturation [61]. Embryos, however, use pyruvate and glucose as energy sources [62]. This change in energy source during development could contribute to the smaller effect of NEFA exposure observed during oocyte maturation as compared to embryo culture. The window of exposure may also determine the extent of DNA methylation dysregulation and regulation of gene expression. DNA methylation increases during oocyte growth and these marks are erased shortly after fertilization, which could provide an opportunity to compensate for aberrant DNA methylation incurred during IVM. This could account for the lower number of differentially methylated loci noticed in blastocysts derived from NEFA-exposed oocytes, compared to blastocysts derived from NEFA-exposed embryos. Salilew-Wondim et al. [54] recently suggested a similar hypothesis as they observed a strong positive association between the extent of DNA methylation dysregulation and the developmental stages completed under in vitro conditions.

Finally, the current study not only helps to unravel the window of epigenetic sensitivity for environmental changes in NEFA concentrations, but also should be integrated to see potential associations between DNA methylation, gene expression and phenotypic profiles. The correlation between canonical pathways in which genes were differentially methylated and expressed was investigated in both IVM and IVC experiments and has been presented in Fig. 11. Overall, pathways affected in both IVM and IVC experiments were mostly related to apoptosis, amino acid metabolism, lipid metabolism and carbohydrate metabolism.

## Conclusions

In the present study, we were only able to acquire an overall perspective of the extent of the differences in methylation. A more in-depth assessment of DNA methylation changes at the level of individual genes was not feasible due to both methodological and biological limitations. Although the EDMA array provides a robust whole-genome view of DNA methylation in bovine early embryos, subtle alterations are not detected through multiple testing corrections. Additionally, vast epigenetic erasure and reprogramming events during early embryogenesis make this phase of development a sensitive window for perturbations. This dynamic window makes the blastocyst both an intriguing and complicated target for further research.

Elevated NEFA concentrations during either in vitro oocyte maturation or embryo development as observed in females suffering metabolic disorders associated with upregulated lipolysis, impact the resultant blastocysts’ transcriptomic and epigenetic profiles. Key cellular pathways affected by NEFA exposure are similar after integration of gene expression and methylation patterns, with particular reference to lipid and carbohydrate metabolism, cell death, immune response and metabolic disorders. Furthermore, early cleavage embryos were even more susceptible to altered in vitro conditions than oocytes during maturation, as a higher level of epigenetic dysregulation was evident in the former. This information is crucial for helping to elucidate the underlying mechanisms of subfertility and development of metabolic disease in the offspring. It suggests that maternal metabolic disorders can influence epigenetic reprogramming in the embryo and affect subsequent development, potentially imprinting long-lasting marks during later stages of adult life. However, more research is necessary to investigate long-term effects of this epigenetic dysregulation.

## Abbreviations

BSA: Bovine serum albumin
COCs: Cumulus oocyte complexes
DMRs: Differentially methylated regions
DOHAD: Developmental origins of adult health and disease
EDMA: EmbryoGENE DNA Methylation Array
FDR: False discovery rate
ICM: Inner cell mass
IPA: Ingenuity Pathways Analysis
IVC: In vitro culture
IVM: In vitro maturation
mEGF: Murine epidermal growth factor
NEFA: Non-esterified fatty acid
OA: Oleic acid
PA: Palmitic acid
SA: Stearic acid
TE: Trophectoderm
